# Identification of Differentially Expressed Genes Induced by Aberrant Methylation in Oral Squamous Cell Carcinomas Using Integrated Bioinformatic Analysis

**DOI:** 10.3390/ijms19061698

**Published:** 2018-06-07

**Authors:** Xiaoqi Zhang, Hao Feng, Dongfang Li, Shanshan Liu, Norio Amizuka, Minqi Li

**Affiliations:** 1 Shandong Provincial Key Laboratory of Oral Tissue Regeneration, Department of Bone Metabolism, School of Stomatology, Shandong University, Jinan 250012, China; zhangxiaoqi67@163.com (X.Z.); kqyxyfh@163.com (H.F.); 15098820559@163.com (D.L.); liushan633423@163.com (S.L.); 2 Department of Developmental Biology of Hard Tissue, Graduate School of Dental Medicine, Hokkaido University, Sapporo 060-8586, Japan; amizuka@den.hokudai.ac.jp

**Keywords:** oral, squamous cell carcinoma, bioinformatics, methylation, differentially expressed genes

## Abstract

Oral squamous cell carcinoma (OSCC) is a malignant disease. Methylation plays a key role in the etiology and pathogenesis of OSCC. The goal of this study was to identify aberrantly methylated differentially expressed genes (DEGs) in OSCCs, and to explore the underlying mechanisms of tumorigenesis by using integrated bioinformatic analysis. Gene expression profiles (GSE30784 and GSE38532) were analyzed using the R software to obtain aberrantly methylated DEGs. Functional enrichment analysis of screened genes was performed using the DAVID software. Protein–protein interaction (PPI) networks were constructed using the STRING database. The cBioPortal software was used to exhibit the alterations of genes. Lastly, we validated the results with the Cancer Genome Atlas (TCGA) data. Twenty-eight upregulated hypomethylated genes and 24 downregulated hypermethylated genes were identified. These genes were enriched in the biological process of regulation in immune response, and were mainly involved in the PI3K-AKT and EMT pathways. Additionally, three upregulated hypomethylated oncogenes and four downregulated hypermethylated tumor suppressor genes (TSGs) were identified. In conclusion, our study indicated possible aberrantly methylated DEGs and pathways in OSCCs, which could improve the understanding of the underlying molecular mechanisms. Aberrantly methylated oncogenes and TSGs may also serve as biomarkers and therapeutic targets for the precise diagnosis and treatment of OSCCs in the future.

## 1. Introduction

Head and neck squamous cell carcinoma (HNSC) is a common malignant disease in the world, accounting for 90% of head and neck cancers. Oral squamous cell carcinoma (OSCC) is the main subtype of HNSCs. OSCCs, characterized by high incidence and mortality, are considered aggressive, based mainly on clinical behavior, with frequent local recurrences, as well as regional and distant metastasis [1]. The incidence of OSCCs is related to smoking, drinking, and viral infections (especially *HPV*), as well as a lack of vitamins and trace elements, such as folic acid, vitamins A, C, and E, zinc, and selenium [2,3,4,5]. The accumulation of many genetic and epigenetic alterations in head and neck epithelial cells are also regarded as central in the initiation and progression of OSCCs [6].

Since the post-genomic era, the study of tumor pathogenesis is no longer confined to gene mutation, gene deletion, etc. In recent years, the role of epigenetics in tumorigenesis has drawn growing attention. Aberrant DNA methylation can influence the occurrence and development of tumors by affecting chromatin structure, and the regulation of oncogenes and tumor suppressor genes at the transcriptional level [7,8]. As an important tumor epigenetic mechanism, DNA methylation was extensively studied with respect to DNA damage repair, cell cycle regulation, angiogenesis, and apoptosis, which are all associated with CpG-island methylation in regulatory regions [9,10]. Abnormal methylation in OSCCs can affect the functions of normal genes, especially the expression of tumor suppressor genes, resulting in the occurrence and development of OSCCs [6]. Although some studies demonstrated certain genes with aberrant DNA hypermethylation or hypomethylation in OSCCs, the comprehensive profiling and pathways of the interactions in this network remain largely elusive.

In recent years, microarrays based on high-throughput platforms unveiled themselves as promising and efficient tools in the search for meaningful genes and epigenetic alternations in carcinogenesis, and in the identification of biomarkers used in diagnosis or prognosis [11]. Gene-expression-profile microarrays were used to find differentially expressed genes (DEGs) in OSCCs [12]. Additionally, many studies on aberrant methylation in OSCCs were performed to find differentially methylated genes (DMGs) [13]. By using integrated and advanced bioinformatic analysis for the available microarray data, more reliable and precise results may be revealed through overlapping relevant datasets.

Until now, there was almost no research that attempted to combine gene-expression-profile microarrays and gene-methylation-profile microarrays to analyze data in the context of the development of OSCCs. In this study, the data from gene-expression-profile microarrays (GSE30784), gene-methylation-profile microarrays (GSE38532), and oncogenes and tumor suppressor genes (TSGs) were integrated and systematically analyzed using bioinformatics. Analyses included obtaining DEGs and DMGs using the R software, overlapping three datasets using a Venn diagram, screening gene-enrichment analysis of gene ontology (GO) and pathway, protein–protein interaction (PPI) network analysis, and the identification and validation of oncogenes and TSGs. We aimed to find aberrantly methylated DEGs, GO, and pathways between tumor groups and normal groups to help uncover the underlying molecular mechanisms of tumorigenesis in OSCCs. We also expected to find novel aberrantly methylated oncogenes or TSGs which might serve as biomarkers and therapeutic targets for the precise diagnosis and treatment of OSCCs.

## 2. Results

### 2.1. Identification of the DEGs and DMGs in OSCCs

**Figure 1:**
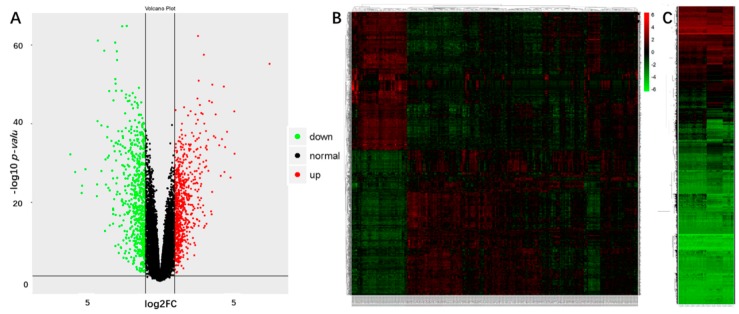
The results of differentially expressed genes (DEGs) in GSE30784, and differentially methylated probes (DMPs) in GSE38532. (**A**) Volcano Plot visualizing the DEGs. The vertical lines demark the fold-change values. The right vertical line corresponds to log2FC > 1 changes, while the left vertical line corresponds to log2FC < −1 changes. The horizontal line marks a −log10 *p*-value of 0.05. (**B**) Heat-map hierarchical clustering revealed 1417 genes that were differentially expressed in oral squamous cell carcinoma (OSCC) groups when compared with control groups. Red and green colors indicate higher expression and lower expression, respectively. (**C**) Heat map of the DMPs. Hierarchical clustering showed separate groupings of DMPs for OSCC tissue and normal tissue.

We got the expression matrices from GSE30784 (containing 167 OSCC samples, and 45 normal samples, GPL570) after data preprocessing and quality assessment using the R software. We set the cut-off criteria as |logFC| > 1, and the *p*-value < 0.05 to screen the DEGs. A total of 1417 DEGs were obtained, including 725 upregulated genes and 692 downregulated genes. Figure 1A,B shows the DEGs using a volcano plot and a hierarchical-clustering heat map. There were 18,119 differentially methylated probes (DMPs) obtained to compare normal samples and tumor samples in GSE38532, as shown in Figure 1C as a heat map. Subsequently, 704 hypermethylated genes and 545 hypomethylated genes were also obtained.

### 2.2. Identification of Aberrantly Methylated Differentially Expressed Genes 

**Figure 2:**
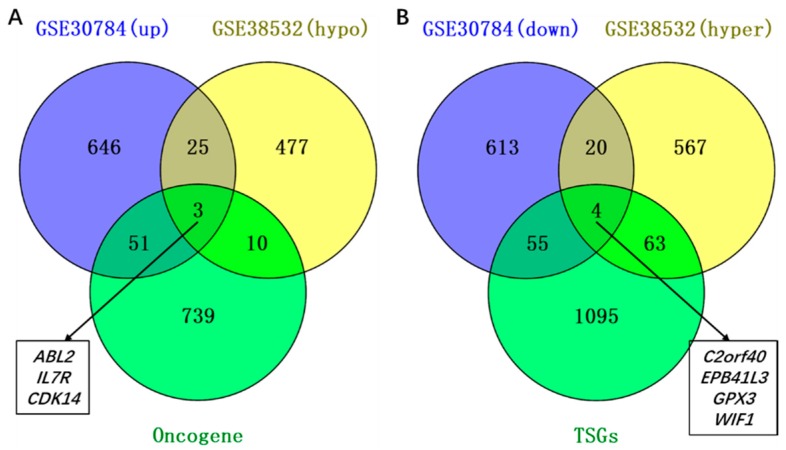
Identification of aberrantly methylated differentially expressed genes, and relating oncogenes and tumor suppressor genes (TSGs). (**A**) Twenty-eight hypomethylated and upregulated genes were identified, and three of them were oncogenes. (**B**) Twenty-four hypermethylated and downregulated genes were identified, and four of them were TSGs.

To explore the aberrantly methylated differentially expressed genes, we overlapped the upregulated genes with the hypomethylated genes, and overlapped the downregulated genes with the hypermethylated genes. We got 28 upregulated hypomethylated genes, and 24 downregulated hypermethylated genes. To further explore the aberrantly methylated DEGs, we overlapped the up-hypomethylated genes with oncogenes, and got three up-hypomethylated oncogenes (*ABL2*, *IL7R*, and *CDK14* in Figure 2A), indicating that the aberrant methylation of those oncogenes led to a high expression in OSCCs, and contributed to tumorigenesis. Meanwhile, by overlapping down-hypermethylated genes with TSGs, four down-hypermethylated TSGs (*C2orf40*, *EPB41L3*, *GPX3*, and *WIF1* in Figure 2B) were obtained, indicating the possibility that aberrant hypermethylation in those TSGs resulted in a low expression in OSCCs, and promoted tumorigenesis. All genes were shown in Table S1.

### 2.3. Functional Enrichment Analysis

**Figure 3:**
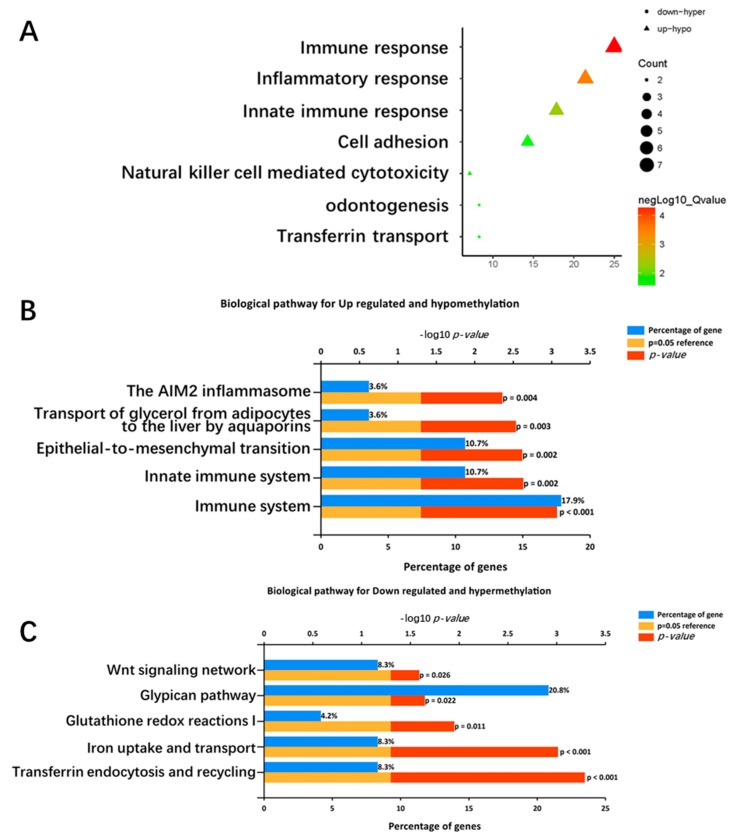
Enrichment analysis of aberrantly methylated differentially expressed genes. (**A**) Significantly enriched biological processes were ranked by *p*-value for the aberrantly methylated differentially expressed genes using the DAVID software. A *p*-value < 0.05 was regarded as significant. (**B**) The top five pathways in which the 28 upregulated hypomethylated genes were significantly involved are shown (ranked by *p*-value using the FunRich 3.0 software). A *p*-value < 0.05 was regarded as significant. (**C**) The top five pathways in which the 24 downregulated hypermethylated genes were significantly involved are shown (ranked by *p*-value using the FunRich 3.0 software). A *p*-value < 0.05 was regarded as significant.

To explore the biological processes and pathways in which the overlapped genes were involved, we uploaded the two lists into the DAVID software and the FunRich software, and obtained the results. A *p*-value < 0.05 was considered significant. Biological-process-enrichment analysis using the DAVID software (Figure 3A) suggested that up-hypomethylated genes were significantly enriched in immune response, inflammatory response, innate immune response, cell adhesion, and natural-killer-cell-mediated cytotoxicity. The down-hypermethylated genes were significantly enriched in odontogenesis and transferrin transport. As for biological pathways (Figure 3B,C), the up-hypomethylated genes were enriched in the immune system, the innate immune system, epithelial to mesenchymal transition, the transport of glycerol from adipocytes to the liver by aquaporins, and the AIM2 inflammasome, while the down-hypermethylated genes were enriched in transferrin endocytosis and recycling, iron uptake and transport, the glypican pathway, and the Wnt signaling network.

### 2.4. PPI Network Construction

**Figure 4:**
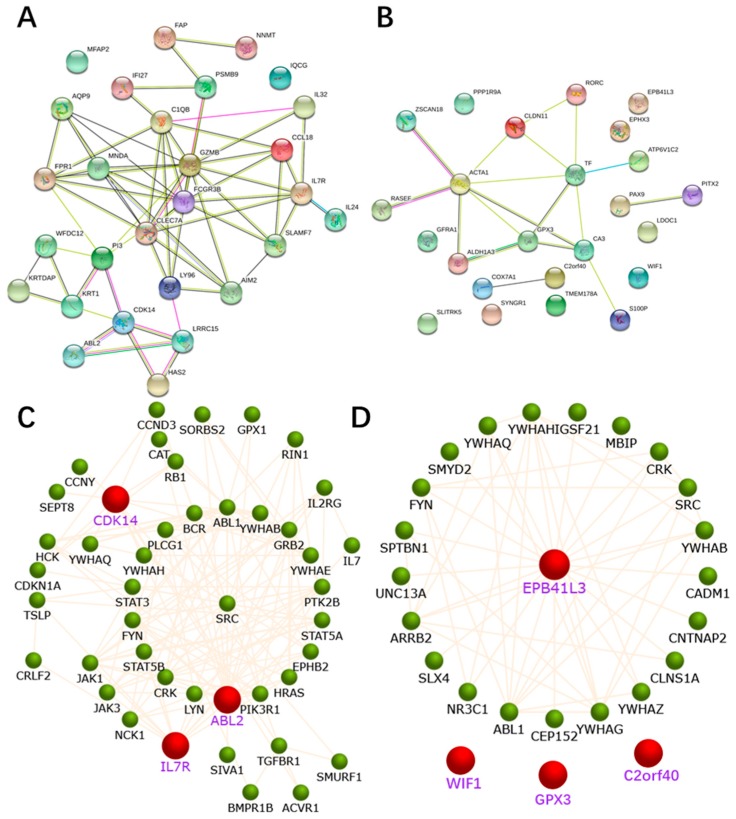
The protein–protein interaction (PPI) network complex for the aberrantly methylated differentially expressed genes. (**A**) A total of 28 genes were filtered into the upregulated hypomethylated PPI network complex using the STRING online database. (**B**) A total of 24 genes were filtered into the downregulated hypermethylated PPI network complex using the STRING online database. (**C**) The PPI network of the three up-hypomethylated oncogenes, and their related genes, created by the FunRich software. (**D**) The PPI network of the four down-hypermethylated TSGs, and their related genes, created by the FunRich software.

We used the online database, STRING, to construct the PPI network. For the 28 upregulated hypomethylated genes, the PPI network contained 28 nodes and 64 edges (Figure 4A). The PPI enrichment *p*-value was 4.44 × 10^−16^ (Figure 4A). The PPI network of the 24 downregulated hypermethylated genes is illustrated in Figure 4B. There were 24 nodes and 18 edges, and the enrichment *p*-value was 3.35 × 10^−3^. Figure 4C,D show the three up-hypomethylated oncogenes, and the four down-hypermethylated TSGs, and their associated genes, allowing us to evaluate their biological functions. Table 1 demonstrates the pathways they were mainly involved in. The STRING version of Figure 4C,D are shown in Figure S1 and Table S2.

### 2.5. Identification and Validation of the Seven Selected Genes

**Figure 5:**
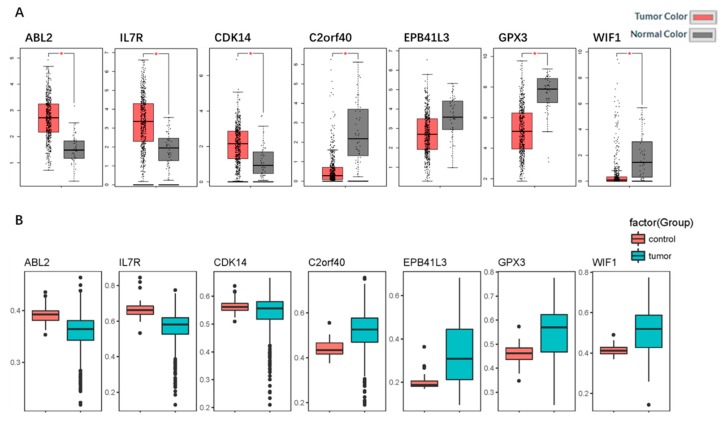
Validation of the seven genes in the Cancer Genome Atlas (TCGA) database. (**A**) Box plots showing the expression of the seven genes in messenger RNA (mRNA) expression, using data from the TCGA database in GEPIA. The statuses of the expression of six genes were the same as in our study, and their *p*-values < 0.05 (*EPB41L3* was not statistically significant). (**B**) Box plots showing the methylation status of the seven genes using the data from the TCGA database. The methylation statuses were in accordance with our study.

**Figure 6:**
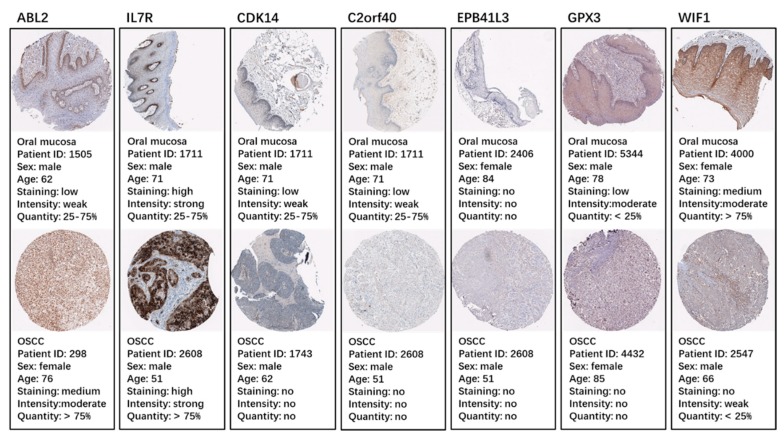
Validation of the seven genes on a translational level using the Human Protein Atlas database (IHC: immunohistochemistry).

To further validate our results, we employed the Cancer Genome Atlas (TCGA) database. Based on the TCGA HNSC data, we found that the expressions of the three up-hypomethylated oncogenes, and the four down-hypermethylated TSGs were significantly different between normal tissues and tumor tissues (Figure 5A). The trend of these seven genes in the TCGA data was the same as observed in our data. Moreover, the immunohistochemistry staining obtained from the Human Protein Atlas database demonstrated the deregulation of the expression of these seven genes (Figure 6). By using the TCGA data for validation, we found that the expression of *EPB41L3* was not significantly different between normal samples and tumor samples. This needs to be confirmed by further experiments. However, data for the remaining six oncogenes/TSGs were in accordance with our data. As for the methylation status of the seven genes, the TCGA HNSC data were used, and the results were found to be the same as our data. The methylation statuses of the seven genes were significantly different between normal samples and tumor samples, and this trend was in accordance with our results.

### 2.6. Genetic Information of the Seven Genes

**Figure 7:**
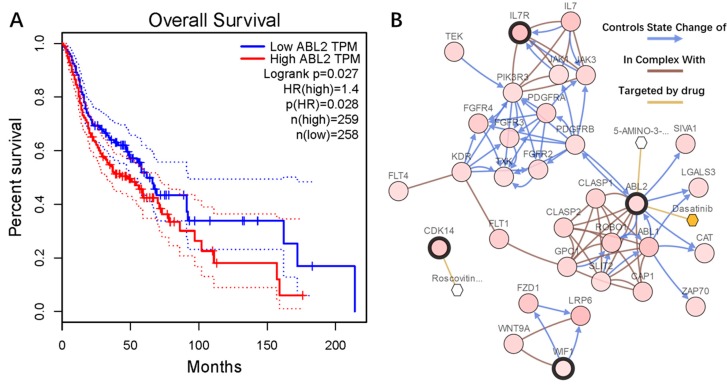
(**A**) *ABL2* was significantly associated with overall survival in head and neck squamous cell carcinoma (HNSC) patients, using a Kaplan–Meier curve and a log-rank test. The patients were stratified into a high-level group and a low-level group according to median. (**B**) The network contained 54 nodes, including our four query genes and the 50 most frequently altered neighbor genes. The relationship between hub genes and drugs is also exhibited.

**Figure 8:**
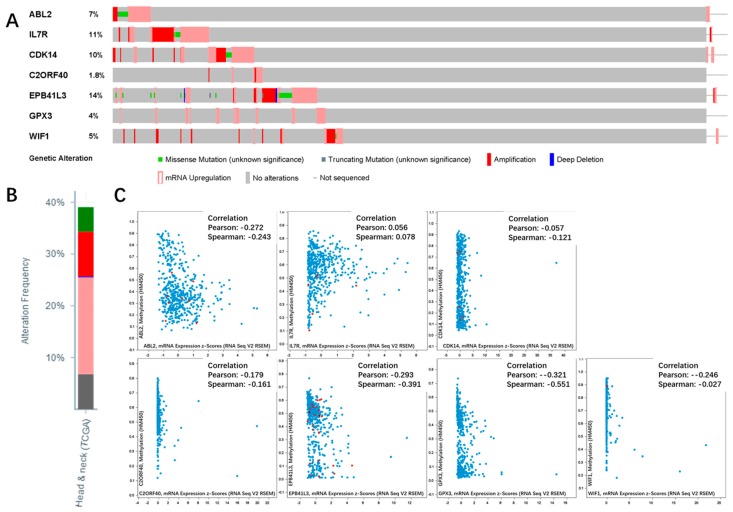
Genetic alterations connected with the seven genes, and the correlation between mRNA and DNA methylation in the TCGA HNSC study. (**A**) A visual summary across a set of HNSC (data from head and neck squamous cell carcinoma TCGA, provisional) shows the genetic alteration of the seven genes which were altered in 207 (41%) of the 510 sequenced cases/patients (528 total). (**B**) An overview of changes in the seven genes in the genomics datasets of TCGA. (**C**) Correlation between mRNA and DNA methylation in the seven genes in TCGA HNSC patients.

We conducted survival analysis using GEPIA to evaluate the relationship between the seven genes and the prognosis. We found that the upregulated hypomethylated oncogene *ABL2* was closely related to overall survival (OS) (Figure 7A). The deregulation of oncogene *ABL2* caused by aberrant methylation could result in poor OS. As for the remaining six genes, the trends were similar with our prognosis, but not statistically significant. The cBioPortal software was employed to explore the genetic alteration of the seven genes. Figure 7B illustrates the network constructed by our seven genes, and their 50 most frequently altered neighbor genes (only three of the seven had a connection node, while the remaining four had no connections, and were not shown). Additionally, drugs targeting the seven genes were exhibited, and only *ABL2* and *CDK14* were currently considered as drug targets. We suppose that the other five genes may serve as novel drug targets in the future. The alteration information of the seven genes is exhibited in Figure 8A,B. We found that the seven genes were altered in 207 (41%) of the 510 sequenced cases/patients (528 total), and that *EPB41L3* and *IL7R* were altered most often (14% and 11%), including amplification, missense, etc. Figure 8C demonstrates the correlation between messenger RNA (mRNA) and DNA methylation of the seven genes in the TCGA HNSC patients. We found that the correlation was negative, indicating that methylation regulated the mRNA expression of these genes (except for *IL7R*). This illustrated that methylation played an important role in the expression of these genes.

## 3. Discussion 

NCBI-GEO is a free database for microarray profiling and next-generation sequencing. The gene-expression-profile dataset (GSE0784), and the gene methylation profile (GSE38532) were obtained there. The R software is a powerful tool for the analysis of microarray data, allowing users to compare different groups of samples, in an effort to screen genes that are differentially expressed through experimental conditions. Studies of the mechanism involved in the cause and development of OSCCs are of great help for the diagnostic, treatment, and prognostic assessment of OSCC patients. In our study, we obtained three upregulated hypomethylated oncogenes, and four downregulated hypermethylated TSGs in OSCCs, using bioinformatic analysis. Functional enrichment of these genes revealed that aberrant methylation indeed affects certain pathways and hub genes. These results can provide novel insight into the explanation of OSCC pathogenesis.

GO analysis demonstrated the enrichment of aberrantly methylated differentially expressed genes in the regulation of the immune response. Tumorigenesis is closely related to immunity [14], as the immune system has the function of identifying and destroying tumor cells; however, the occurrence and development of malignant tumors is often caused by tumor cells which evade the immune response through immune escape mechanisms. Tumor immune evasion (i.e., tumors making the host body unable to produce an effective anti-tumor immune response through various methods) and its mechanisms are quite complex [15]. Among them, the immunosuppressive cell subsets negatively regulate the anti-tumor immune response, and are an important cause of tumor escape. These subsets include regulatory T cells (Treg), immature dendritic cells (DC), and so on. Pathway enrichment analyses were also found to be concentrated in the regulation of the immune system, as described above.

The PPI network of upregulated hypomethylated oncogenes was constructed using the FunRich software, and was visualized as a network. In this network, the function analysis of these oncogenes was revealed. Involvement in the PI3K-AKT-mTOR pathway was one such function, which was identified as an important pathway in cancer [16]. The mTOR protein is a key kinase downstream of the PI3K/AKT proteins, which regulate cancer cell proliferation, growth, survival, and angiogenesis [17]. Cancer cells can escape normal biochemical systems that keep the balance between apoptosis and survival. The PI3K-AKT-mTOR pathway generally functions by promoting survival through the inhibition of proapoptotic factors, and the activation of anti-apoptotic factors. Through phosphorylation, the PI3K-AKT-mTOR pathway inhibits the activity of proapoptotic members while activating anti-apoptotic members. To negatively regulate PI3K, cells contain the *PTEN* phosphatase [18]. A reduction in PTEN expression indirectly stimulates the PI3K-AKT-mTOR pathway’s activity, thereby contributing to oncogenesis in humans. Recent data suggested that the PI3K-AKT-mTOR signaling pathway plays an important role in cancer stem-cell self-renewal, and the resistance to chemotherapy or radiotherapy [19], believed to be the root of treatment failure and cancer recurrence, as well as metastasis. From these results, we can understand the importance of the function of these modules in HNSC, and recognize the need for further investigation to confirm these results.

The three upregulated hypomethylated oncogenes were *ABL2*, *IL7R*, and *CDK14*. *ABL2* encodes a member of the Abelson family of nonreceptor tyrosine protein kinases. The protein is highly similar to the *c-abl* oncogene 1 protein, and it plays a role in cytoskeletal rearrangement through its C-terminal F-actin- and microtubule-binding sequences [20]. This gene is expressed in both normal and tumor cells, and is involved in the translocation of the *ETV6* gene in leukemia [21]. It was reported that high expression of *ABL2* results in a poor prognosis in hepatocellular carcinomas, and the overexpression of *ABL2* can promote cancer cell migration and invasion [22]. Similar reports, concerned with prostate cancer and breast cancer, also reported that *ABL2* promotes cancer cells invasion and migration [23,24]. However, the role of *ABL2* in OSCCs is currently yet to be determined. From our results, we found that *ABL2* was indeed highly expressed in OSCC tissues when compared with normal samples from multiple datasets. The survival analysis results demonstrated that *ABL2* was closely related with OS, indicating that *ABL2* could be a predictor of poor prognosis in OSCC patients. Furthermore, aberrant methylation may be the reason for the high expression of *ABL2* in OSCC, which is also yet to be reported. The rate of *ABL2* mutation was 7% in HNSCs, and we suppose that the mutation resulted in the aberrant methylation or deregulation of *ABL2*. *ABL2* is only the target for one kind of drug at present, and *ABL2* may serve as a drug target for more drugs in the future, such as anti-tumor drugs. The protein encoded by *IL7R* is a receptor for interleukin 7 (IL7). This protein was shown to play a critical role in V(D)J recombination during lymphocyte development [25]. Defects of this gene may be associated with severe combined immunodeficiency (SCID) [26]. It may also play an important role in tumor immune evasion. The reports on *IL7R* were mainly concerned with its mutational activation, which is involved in human T-cell leukemogenesis [27]. In HNSCs, *IL7R* was found to be altered about 11% of the time, indicating a similar mechanism may exist in OSCC tumorigenesis. It was reported that T cells lack the expression of IL7 receptor α (IL7Rα), which is associated with the hypermethylation of the IL7R promoter, therefore restricting T-cell development in SIOD patients [28]. From our results, we found that hypomethylation in *IL7R* led to a high expression in OSCC samples, which may affect the anti-tumor immune response during tumorigenesis. The *CDK14* protein is a member of the *CDC2* (MIM 116940)-related protein kinase family, and acts as a regulator of cell cycle progression and cell proliferation via its interaction with *CCDN3* [29]. It was reported that a high expression of CDK14 was associated with poor prognosis in osteosarcoma, and *miR-216a* was found to target CDK14, resulting in the inhibition of cell proliferation, invasion, and metastasis of osteosarcoma [30]. In our study, we found that *CDK14* affected by hypomethylation resulted in a high expression in OSCCs, indicating that *CDK14* may function as a promoter of OSCC proliferation. *CDK14* was altered in about 10% of HNSC patients, and we suppose that mutations in *CDK14* led to deregulation in OSCCs. Reports on breast cancer demonstrated that an allele-specific copy-number imbalance in *CDK14* was related to poor prognosis [31]. The three oncogenes we found were highly expressed in OSCC samples, and we suppose that they play an important role in OSCC tumorigenesis. Aberrant methylation of these three oncogenes may be the reason for their deregulation in OSCCs. 

The four downregulated hypermethylated TSGs were *C2orf40*, *EPB41L3*, *GPX3*, and *WIF1*. *C2orf40* (chromosome 2 open reading frame 40) is a protein-coding gene, associated with diseases such as esophageal cancer [32]. It functions as a probable hormone that induces senescence of oligodendrocyte and neural precursor cells, characterized by G1 arrest, *RB1* dephosphorylation, and accelerated *CCND1* and *CCND3* proteasomal degradation [33]. *EPB41L3* is a tumor suppressor that inhibits cell proliferation, and promotes apoptosis [34]. It also modulates the activity of protein arginine N-methyltransferases, including *PRMT3* and *PRMT5*. It was widely reported for multiple cancers, such as esophageal cancer and hepatocellular carcinoma, that a high expression of *EPB41L3* in tumor cells promotes migration and invasion, and is related with poor prognosis [35,36]. The protein encoded by *GPX3* belongs to the glutathione peroxidase family, members of which catalyze the reduction of organic hydroperoxides, and hydrogen peroxide (H_2_O_2_) by glutathione, thereby protecting cells against oxidative damage [37]. Downregulation of the expression of this gene through the promotion of hypermethylation was observed in a wide spectrum of human malignant diseases, including thyroid cancer, hepatocellular carcinoma, and chronic myeloid leukemia. A low expression of *GPX3* was found to be a biomarker for poor prognosis in gallbladder cancer, and silencing *GPX3* was found to promote tumor metastasis of thyroid cancer in which *GPX3* functioned as a TSG [38,39]. *WIF1* functions to inhibit Wnt proteins, which are extracellular signaling molecules that play a role in embryonic development [40]. It also functions as a tumor suppressor gene, and was found to be epigenetically silenced in various cancers. A downregulation of *WIF1* was widely reported for multiple cancers, such as prostate cancer, breast cancer, lung cancer, and bladder cancer, and was correlated with a more advanced tumor stage [41]. It functions as an inhibitor of the Wnt signaling pathway, and could regulate *SKP2* and *c-Myc* expression, resulting in G1 arrest and the inhibition of proliferation in urinary bladder cancer cells [42]. The roles of the four TSGs were not reported for OSCCs. In our study, we found that they were hypermethylated and downregulated in OSCCs, indicating that aberrant methylation in OSCCs might lead to the deregulation of these TSGs, resulting in OSCC tumorigenesis.

We acknowledge that there were some limitations and shortcomings in this study. Firstly, we focused on the upregulated hypomethylated and downregulated hypermethylated genes without analyzing the contra-regulated genes. Further analysis considering this aspect is needed in the future. Secondly, the clinical parameters and prognoses were not analyzed due to the availability of data. The validation of aberrantly methylated genes was carried out using TCGA data, and further experiments are needed to produce a solid confirmation of these results in relation to OSCCs. 

## 4. Materials and Methods

### 4.1. Microarray Data Information

NCBI-GEO is a free database for microarray/gene profiling and next-generation sequencing. In this study, the gene-expression-profiling dataset (GSE30784) and the gene-methylation-profile dataset (GSE38532) were obtained from the Gene Expression Omnibus (GEO, https://www.ncbi.nlm.nih.gov/geo/) of NCBI. The microarray data from GSE30784 were based on GPL570 Platforms ((HG-U133_Plus_2) Affymetrix Human Genome U133 Plus 2.0 Array; Thermo Fisher Scientific, Waltham, MA, USA), and included 167 OSCC samples and 45 normal samples. The gene-methylation-profile microarray data from GSE33202 were based on GPL8490 Platforms (Illumina HumanMethylation27 BeadChip (HumanMethylation27_270596_v.1.2; Thermo Fisher Scientific), and contained 40 OSCC samples and 40 normal samples.

### 4.2. Data Processing for the Identification of DEGs and DMGs

We used the R software (version 3.4.3; Bell Laboratories, formerly AT&T, now Lucent Technologies, Murray Hill, NJ, USA) to analyze GSE30784 and GSE38532, in an effort to identify DEGs and DMGs. For the DEGs, we set cut-off criteria as *p*-value < 0.05, and logFC (>1 or <−1). As for the DMPs, we set FDR < 0.05 and cut-off β > 0.2. The computer code is provided in the Supplementary Materials (in the “computer code” section). We then transformed the DMG identifiers (IDs) to gene names using the wANNOVAR tool (http://wannovar.wglab.org/). We got the oncogene list from the ONGene database (http://ongene.bioinfo-minzhao.org/), and the TSG list from the TSGene database (https://bioinfo.uth.edu/TSGene/index.html). Next, we used an online Venn diagram tool (http://bioinfogp.cnb.csic.es/tools/venny/) to identify overlapping DEGs, DMGs, oncogenes, and TSGs. Lastly, we obtained upregulated hypomethylated oncogenes by overlapping hypomethylated genes, upregulated genes, and oncogenes. The same method was used to obtain downregulated hypermethylated genes, overlapping hypermethylated genes, downregulated genes, and TSGs.

### 4.3. Gene Ontology and Pathway Enrichment Analysis

The Database for Annotation, Visualization, and Integrated Discovery (DAVID, https://david.ncifcrf.gov/) was employed to perform the gene ontology enrichment analysis. We submitted our lists, which contained 28 upregulated hypomethylated genes and 24 downregulated hypermethylated genes, into DAVID. A *p*-value < 0.05 was regarded as statistically significant, and the GO results were ranked by *p*-value. The significant terms for biological processes (BPs) were selected. The functional enrichment analysis tool (FunRich, version: FunRich 3.0, http://www.funrich.org/) is a stand-alone software tool used mainly for functional enrichment and interaction network analysis of genes and proteins. The FunRich tool was used to unravel the pathways behind the genes we submitted. A *p*-value < 0.05 was regarded as statistically significant, and the top five significant pathways in the upregulated hypomethylated genes and the downregulated hypermethylated genes were exhibited as bar charts. 

### 4.4. Protein–Protein Interaction (PPI) Network Construction and Module Analysis

Protein–protein interaction (PPI) analysis is important for the interpretation of molecular mechanisms of the key cellular activities in carcinogenesis. In our study, the Search Tool for the Retrieval of Interacting Genes (STRING) database (https://string-db.org/cgi/input.pl) and the FunRich tool were employed to construct the PPI network. The functional enrichment analyses of the up-hypomethylated oncogenes and the down-hypermethylated TSGs were carried out in FunRich, and we selected the top five pathways for each group.

### 4.5. Validation of the Seven Genes 

The Cancer Genome Atlas (TCGA) database includes comprehensive, multi-dimensional maps of key genomic changes in various types of cancers. In order to confirm our results, we validated the seven oncogenes/TSGs using TCGA data. We downloaded the TCGA data from UCSC Xena (http://xena.ucsc.edu/). The expressions of the seven oncogenes/TSGs were compared between normal samples and OSCC samples. Furthermore, the validation of the translational levels of the seven oncogenes/TSGs was carried out using the Human Protein Atlas database (https://www.proteinatlas.org/). The IHC (immunohistochemistry) pathological section between the normal and OSCC samples was used to validate the results. The cBio Cancer Genomics Portal (http://www.cbioportal.org/) is an open platform, providing visualization, analysis, and downloads of large-scale cancer genomics datasets of various cancers. Complex cancer genomics profiles are easily obtained using the query interface of the portal, enabling researchers to explore and compare genetic alterations across samples. We used the cBioPortal tool to explore the genetic alterations connected with the seven genes, and the correlation between mRNA and DNA methylation in HNSC studies.

## 5. Conclusions

Our results identified aberrantly methylated and differentially expressed oncogenes and TSGs, and their related pathways and functions by means of integrated bioinformatic analysis. These results may contribute to uncovering the molecular mechanisms underlying the initiation and development of OSCCs. The seven genes found were validated using the TCGA, and included *IL7R*, *CDK14*, *ABL2*, *EPB41L3*, *GPX3*, *WIF1*, and *C2orf40*. These genes might serve as aberrant methylation-based biomarkers and therapeutic targets for the precise diagnosis and treatment of OSCCs in the future. When compared with single-dataset investigations, our study provided more reliable and accurate results by using several datasets. Further experiments are needed to confirm the candidate genes which we uncovered in OSCCs.

## Figures and Tables

**Table 1:** The pathways in which the three oncogenes and the four tumor suppressor genes (TSGs) were mainly involved.

#	Node	Count	*p*-Value
**Upregulated and hypomethylated oncogene**			
1	Proteoglycan syndecan-mediated signaling events	32	*p* < 0.001
2	Class I PI3K signaling events	31	*p* < 0.001
3	Insulin Pathway	31	*p* < 0.001
4	mTOR signaling pathway	31	*p* < 0.001
5	Class I PI3K signaling events mediated by AKT	31	*p* < 0.001
**Downregulated and hypermethylated TSGs**			
1	α6β4 integrin	7	*p* < 0.001
2	Neurotrophic factor-mediated Trk receptor signaling	8	*p* < 0.001
3	p75(NTR)-mediated signaling	9	*p* < 0.001
4	Regulation of nuclear SMAD2/3 signaling	10	*p* < 0.001
5	TGF-β receptor signaling	10	*p* < 0.001

## Supplementary Materials

The following are available online at http://www.mdpi.com/1422-0067/19/6/1698/s1.

## Abbreviations

HNSC	Head and neck squamous cell carcinoma
DEGs	Differentially methylated genes
DMGS	Differentially methylated genes
PPI	Protein–protein interaction
GO	Gene ontology
BP	Biological process
CC	Cell component
MF	Molecular function
