# Supplementary material for: Identification of Differentially Expressed Genes Induced by Aberrant Methylation in Oral Squamous Cell Carcinomas Using Integrated Bioinformatic Analysis
Source: Int J Mol Sci. 2018 Jun 7;19(6):1698. doi: 10.3390/ijms19061698 (PMC6032197; doi:10.3390/ijms19061698)
Supplement: Supplementary file 1 [file ijms-19-01698-s001.pdf]

Table S1. The 28 hypomethylated/upregulated genes and the 24 hypermethylated/downregulated genes.

Upregulated and hypomethylated genes
FAP, MFAP2, NNMT, IFI27, LRRC15, CLEC7A, C1QB, FCGR3B, AIM2, FPR1, IL24, IL32, LY96, PSMB9, GZMB, CCL18, IQCG, MNDA, HAS2, AQP9, PI3, SLAMF7, WFDC12, KRT1, KRTDAP, ABL2, IL7R, CDK14
Downregulated and hypermethylated genes
CLDN11, PAX9, SYNGR1, SLITRK5, GFRA1, RORC, ZSCAN18, LDOC1, TF, EPHX3, PPP1R9A, ATP6V1C2, COX7A1, ALDH1A3, CA3, RASEF, TMEM178A, PITX2, ACTA1, S100P, C2orf40, EPB41L3, GPX3, WIF1

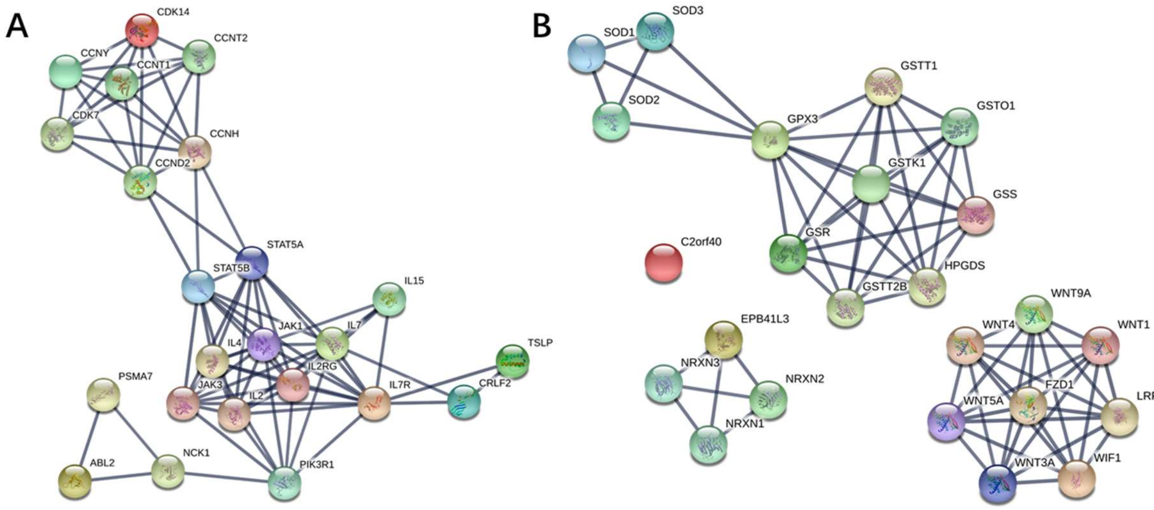

Figure S1. (A) The PPI network of the three upregulated hypomethylated oncogenes, and their related genes created using the STRING database. (B) The PPI network of the four downregulated hypermethylated TSGs, and their related genes created using the STRING database.

Table S2. The pathways in which the three oncogenes and the four TSGs were mainly involved.

#	Node	Count	FDR
Upregulated and hypomethylated oncogenes			
1	Jak-STAT signaling pathway	13	4.58e-20
2	Measles	8	1.3e-10
3	HTLV-I infection	8	1.78e-08
4	Cytokine–cytokine receptor interaction	8	1.82e-08
5	PI3K-Akt signaling pathway	8	1.11e-07
Downregulated and hypermethylated TSGs			
1	Glutathione metabolism	7	2.78e-11
2	Wnt signaling pathway	8	2.82e-10
3	Basal cell carcinoma	6	3.54e-09
4	Melanogenesis	6	8.22e-08
5	Hedgehog signaling pathway	5	1.67e-07

Computer code
DEGs

```
library(affy)
library(limma)
##import phenotype data
phenoData = read.AnnotatedDataFrame('fenzu.txt')
pheno = pData(phenoData)
View(pheno)

##RMA normalization
eset.rma <- justRMA(filenames=paste(rownames(pheno),'.CEL',sep=''))
datExpr = exprs(eset.rma)
##Complementing missing data
library(impute)
##KNN compute missing data
imputed_gene_exp = impute.knn(datExpr,k=10,rowmax = 0.5,
                              colmax=0.8,maxp =3000, rng.seed=362436069)
datExpr2 = imputed_gene_exp$data

######
write.table(datExpr2,file="Expdata2.txt",sep="\t")

#######
##divide groups
Group = factor(pheno$group,levels=c('tumor','control'))
design = model.matrix(~-1+Group)
colnames(design) <- c('tumor','control')
design
## Linear model fitting
fit <- lmFit(datExpr2, design)
# #Build comparison models and compare expression data under two conditions
contrast.matrix <- makeContrasts("tumor-control",
                                 levels=design)

fit1 <- contrasts.fit(fit, contrast.matrix)
## Bayesian test
fit2 <- eBayes(fit1)

allgene<-topTable(fit2,coef="tumor-control",n=nrow(fit2),sort.by="p")
## Generating test table for all genes
biocLite("annotate")
##Download annotate package for annotation
library(annotate)
## Loading annotate package
affydb<-annPkgName(eset.rma@annotation,type="db")
##Get the model of the affy chip we use and store it in the variable affydb
affydb
##View the chip model
#biocLite("hgu133plus2.db")
## According to the chip model, download the chip note package
library(affydb,character.only=TRUE)
allgene$symbols<-getSYMBOL(rownames(allgene),affydb)
```

```
## Obtain the symbol number corresponding to the chip probe
allgene$EntrezID<-getEG(rownames(allgene),affydb)
## Obtain the EntrezID gene number corresponding to the chip probe
write.csv(allgene,file="allgene.csv",quote=F)
allgene<-na.omit(allgene)
## Delete the probe without the corresponding gene number data
EGU<-unique(allgene$EntrezID)
## Select individual genes, repeat genes retain the first small P value, and leave duplicate genes not
to
allgene<-allgene[match(EGU,allgene$EntrezID),]
##Use the match function to pick out test reports for individual genes and keep duplicate genes
allgene$type<-NA
for(i in 1:nrow(allgene))if(allgene[i,1]>1&allgene[i,4]<0.05){allgene[i,9]<-"up"}else
if(-allgene[i,1]>1&allgene[i,4]<0.05){allgene[i,9]<-"down"}else{allgene[i,9]<-"normal"}
## The gene types were divided into three categories by loop statements (logFC>1, p.Val<0.05 for
up-regulated genes up, logFC<-1, p.Val<0.01 for down-regulation genes down, others are normal )
write.csv(allgene,file="allgene2.csv",quote=F)
gene_up<-allgene[allgene$type=="up",]
gene_down<-allgene[allgene$type=="down",]
gene_dif<-allgene[allgene$type!="normal",]
write.csv(gene_up,file="gene_up.csv",quote=F)
write.csv(gene_down,file="gene_down.csv",quote=F)
write.csv(gene_dif,file="gene_dif.csv",quote=F)
install.packages("ggplot2")
library(ggplot2)
threshold<-factor(allgene$type)
pdf('volcano.pdf',onefile=TRUE,width=10,height=10)
ggplot(allgene,aes(x=logFC,y=-log10(P.Value),colour=threshold))+xlab("log2FC")+ylab("-log10P-Val
ue")+ggtitle("Volcano
Plot")+theme(plot.title=element_text(hjust=0.5))+geom_point()+geom_vline(xintercept =
c(-1,1))+geom_hline(yintercept = -log10(0.05))+theme(panel.grid
=element_blank())+scale_colour_manual(values=c("green", "black", "red"))+expand_limits(x=c(-8,8))
## Drawing a volcano with ggplot
dev.off()
biocLite("pheatmap")
library(pheatmap)
gene_dif_exp<-datExpr2[rownames(gene_dif),]
rownames(gene_dif_exp)<-gene_dif$symbols
write.csv(gene_dif_exp,file="gene_dif_exp.csv",quote=F)
tiff(file="gene_dif_pheatmap.tif",res=300,units='in',width=30,height=30)
#pdf('gene_dif_pheatmap.pdf',onefile=TRUE,width=40,height=60)
pheatmap(gene_dif_exp,color=colorRampPalette(c("green","black","red"))(100),fontsize_row=4,scale
="row",border_color=NA)
## Draw a heat map
dev.off()
```

DMPs

```
source("http://bioconductor.org/biocLite.R")
biocLite("minfi")
biocLite("impute")
biocLite("wateRmelon")
install.packages("gplots")
```

```
install.packages("cluster")

library("minfi")
library("impute")
library("wateRmelon")
setwd("C:/Users/y/Desktop/methylation")
info=read.table("group.txt",sep="\t",header=T)
rt=read.table("data.txt",sep="\t",header=T, row.names=1, blank.lines.skip = FALSE)
mat=as.matrix(rt)
mat=impute.knn(mat)
matData=mat$data
matData=matData+0.00001
##normalization
matData=matData[rowMeans(matData)>0.005,]
pdf(file="rawBox.pdf")
boxplot(matData,col = "blue",xaxt = "n",outline = F)
dev.off()
matData = betaqn(matData)
pdf(file="normalBox.pdf")
boxplot(matData,col = "red",xaxt = "n",outline = F)
dev.off()
write.table(matData,file="norm.xls",sep="\t",quote=F)
grset=makeGenomicRatioSetFromMatrix(matData,what="Beta")
##QC
pdf(file="densityBeanPlot.pdf")
par(oma=c(2,10,2,2))
densityBeanPlot(matData, sampGroups = info$Group, sampNames = info$Sample)
dev.off()
pdf(file="mdsPlot.pdf")
mdsPlot(matData, numPositions = 1000, sampGroups = info$Group, sampNames = info$Sample)
dev.off()
##Finding differentially methylated positions (DMPs)
M = getM(grset)
dmp <- dmpFinder(M, pheno=info$Group, type="categorical")
dmpDiff=dmp[(dmp$qval<0.05) & (is.na(dmp$qval)==F),]
write.table(dmpDiff,file="dmpDiff.xls",sep="\t",quote=F)
##heatmap
diffM <- M[rownames(dmpDiff),]
hmExp=diffM
library('gplots')
hmMat=as.matrix(hmExp)
pdf(file="heatmap.pdf",height=150,width=30)
par(oma=c(3,3,3,5))
heatmap.2(hmMat,col='greenred',trace="none",cexCol=1)
dev.off()

##differentially regions
class <- info$Group
designMatrix <- model.matrix(~factor(class))
colnames(designMatrix) <- c("T","C")
dmrs <- bumphunter(grset, design = designMatrix, cutoff = 0.2, B=10, type="Beta")
write.table(dmrs$table,file="dmrs.xls",sep="\t",quote=F)
```
